# Machine learning in keratoconus: severity grading, progression prediction, and surgery outcomes

**DOI:** 10.3389/fcell.2026.1969175

**Published:** 2026-10-21

**Authors:** Junqi Liang, Zijun Zhang, Ahyan Ilman Qudsi, Qingfeng Liang

**Affiliations:** 1 University College London Hospital, London, United Kingdom; 2 Beijing Institute of Ophthalmology, Beijing Tongren Eye Center, Beijing Tongren Hospital, Capital Medical University, Beijing Key Laboratory of Ophthalmology and Visual Sciences, Beijing, China

**Keywords:** keratoconus, artificial intelligence, machine learning, deep learning, disease progression, disease grading

## Abstract

**Aim:**

To review and evaluate machine learning (ML) applications in keratoconus (KC), with a focus on severity classification, progression prediction, and surgical outcome prediction.

**Methods:**

A systematic literature search was conducted in PubMed, Scopus, Web of Science, IEEE Xplore, and Google Scholar for English-language studies published between January 2015 and October 2025. Studies applying ML to KC grading, progression prediction, or surgical outcome prediction were included. Data on clinical tasks, imaging modalities, model architectures, validation strategies, and performance were extracted and evaluated.

**Results:**

Forty-one reports were identified, including 20 on severity classification, 10 on progression assessment, and 11 on surgical outcome prediction. Severity models mainly reproduced clinical or device-derived grades, while progression models addressed different endpoints, including tomographic change and subsequent corneal cross-linking. Surgical outcome models predicted optical changes, treatment response or graft outcomes at different follow-up intervals.

**Conclusion:**

ML showed promising internal performance for KC severity classification, while evidence for progression and surgical outcome prediction remains limited. Clinical translation will require standardized endpoints, patient-separated evaluation, multicenter external validation, calibration assessment, and direct comparison with existing clinical decision rules.

## 1 Introduction

Keratoconus (KC) is the most common primary corneal ectasia (Garcia-Ferrer et al., 2019), characterized by progressive stromal thinning, corneal protrusion, and irregular myopic astigmatism (Niazi et al., 2023). Estimates of its prevalence vary across studies: a meta-analysis reported a pooled prevalence of 1.38 per 1,000 (Hashemi et al., 2020), whereas a meta-analysis estimated 2.89 per 1,000 (Sriranganathan et al., 2026). In the latter analysis, prevalence was highest among adults aged 20–29 years, at 5.26 per 1,000 (Sriranganathan et al., 2026), highlighting the burden of KC during education and early working life. Advances in corneal topography and tomography have improved the detection of ectatic changes (Ambrósio et al., 2023), while corneal collagen cross-linking (CXL) has improved disease management by limiting disease progression and reducing the need for corneal transplantation (Chan, 2020).

Despite these advances, important challenges remain in assessing disease severity, identifying progression, and planning treatment. The course of KC varies between patients, and differences in the definitions and thresholds used to identify progression further complicate its assessment (Gil et al., 2025; Henriquez et al., 2025). Maximum keratometry (Kmax) is a commonly used measure (Henriquez et al., 2025), but its sensitivity for detecting progression depends on the threshold and reference definition applied (Gil et al., 2025). Clinical staging systems, such as the Amsler–Krumeich classification, provide a framework for describing disease severity (Krumeich et al., 1998), although they do not capture all aspects of progression or visual function (Gomes et al., 2015). Clinical decisions therefore require more than a severity label: clinicians must also assess the likelihood of further progression and the expected outcomes of treatment. Decisions about monitoring intervals, the timing of CXL, and surgical treatment currently require the combined interpretation of tomographic parameters, refractive findings, and visual acuity. Whether individualized prediction models can improve these decisions and subsequent patient outcomes remains uncertain.

Machine learning (ML) offers methods for learning patterns from data to support tasks such as classification and prediction. Deep learning (DL), a subset of ML based on multilayer neural networks, is particularly suited to image analysis. The central role of corneal imaging in KC assessment has prompted growing interest in ML and DL for this disease. Recent reviews report high cross-sectional performance for models designed to detect established and subclinical KC (Bodmer et al., 2024; Goodman and Zhu, 2024; Muhsin et al., 2025a). These models use corneal topographic and tomographic maps, measurements derived from Scheimpflug or slit-scanning systems, and anterior-segment optical coherence tomography (AS-OCT) images. High diagnostic performance alone, however, does not establish the value of these methods for disease monitoring or treatment planning. These applications require a distinction between describing current disease severity, identifying change over time, and predicting future progression or treatment outcomes. Much of the available evidence comes from retrospective, single-center studies with small datasets and limited external validation. Differences in outcome definitions, input data, and validation strategies also complicate comparisons across studies.

This review therefore examines ML and DL applications in three areas of KC management: severity classification, progression assessment and prediction, and surgical outcome prediction. For each area, we evaluate the clinical task, data sources and representations, outcome definitions, modeling approaches, and validation methods.

## 2 Methods

### 2.1 Databases and search strategy

A comprehensive literature search was conducted to identify studies applying ML techniques to keratoconus, with particular emphasis on imaging analysis, disease prognosis, and personalized therapeutic approaches. The search strategy was designed in accordance with the Preferred Reporting Items for Systematic Reviews and Meta-Analyses (PRISMA) guidelines (Page et al., 2021) to ensure transparency and reproducibility. Electronic databases including PubMed, Scopus, Web of Science, IEEE Xplore, and Google Scholar were systematically searched. The search covered publications from January 2015 to October 2025. Only studies published in English were included to maintain consistent terminology and ensure clear interpretation of methodological details.

The search strategy combined controlled vocabulary (MeSH terms) and free-text keywords related to both keratoconus and artificial intelligence. The core search string used for PubMed was (“keratoconus” OR “corneal ectasia”) AND (“deep learning” OR “artificial intelligence” OR “machine learning” OR “neural network” OR “transformer” OR “AI”).

### 2.2 Study selection and evidence reconciliation

References from all databases were organized and de-duplicated in Zotero. Two investigators (J.L., Z.Z.) independently screened titles and abstracts using predefined inclusion criteria. Equivalent search terms were adapted for each database, and reference lists of relevant articles and recent reviews were manually screened to ensure comprehensive coverage.

Eligible studies included original research using ML methods for KC grading, progression prediction, and surgical outcome prediction; employed imaging modalities such as corneal topography, tomography, OCT and Scheimpflug imaging; and provided sufficient methodological detail on model architecture, dataset characteristics, and validation. Studies were excluded if they were reviewing articles, conference abstracts without quantitative results, focused solely on classical computer vision methods, or unrelated to KC.

After excluding unrelated references, full texts were reviewed based on these criteria: (1) clear KC task definition; (2) use of ML methods; (3) detailed performance parameters; and (4) completeness of data. Each criterion was scored, with studies scoring 4 were considered high quality. Screening and quality assessment were conducted independently by two researchers (J.L., Z.Z.), with discrepancies resolved by a third (Q.L.).

### 2.3 Data extraction

Data extraction included study objectives, task definition, imaging modality, model architecture, dataset size, validation strategy, performance metrics, and key conclusions. External validation and interpretability analyses were also recorded. The final dataset comprised peer-reviewed studies from ophthalmology, biomedical engineering, and computational imaging journals, ensuring balanced clinical and technical representation. All the data above were extracted into Microsoft Excel.

## 3 Results

A total of 41 reports were included across the three predefined categories: 20 on keratoconus severity classification, 10 on progression assessment or prognosis, and 11 on prediction of outcomes after KC treatment. Across these studies, data sources and assessment modalities included Scheimpflug tomography (mainly Pentacam), Placido-based or combined topography systems (e.g., SCHWIND, TMS-4), corneal biomechanical assessment (Corvis ST), AS-OCT, and frontal or lateral corneal images, supplemented in some surgery-outcome work by Orbscan-derived parameters. The modeling approaches ranged from conventional machine-learning algorithms (e.g., logistic regression, support vector machines, random forests, discriminant analysis, clustering methods) to deep convolutional neural networks (CNNs), recurrent neural networks, time-delay neural networks, variational autoencoder–based clustering, and image-to-image prediction models.

### 3.1 Keratoconus severity classification

#### 3.1.1 Study characteristics and task definitions

Twenty reports evaluated ML methods for keratoconus severity classification or characterization (Table 1). Classification models used several types of ocular data (Hallett et al., 2020; Chen et al., 2021; Herber et al., 2021; Kamiya et al., 2021b; Mahmoud and Mengash, 2021; Malyugin et al., 2021; Shashank et al., 2021; Alyasseri et al., 2022; Ambalgekar and Ashwini, 2023; Dhinakaran et al., 2023; Gao et al., 2023; Muhsin et al., 2024). Pentacam-based datasets were reported by Muhsin et al., Dhinakaran et al., Alyasseri et al., Chen et al., Malyugin et al., and Hallett et al. (Hallett et al., 2020; Chen et al., 2021; Malyugin et al., 2021; Alyasseri et al., 2022; Dhinakaran et al., 2023; Muhsin et al., 2024). Gao et al. used SCHWIND-derived topographic maps (Gao et al., 2023), Ambalgekar et al. and Shashank et al. analyzed corneal topography images from unidentified devices (Shashank et al., 2021; Ambalgekar and Ashwini, 2023), Kamiya et al. used a TMS-4 absolute axial map (Kamiya et al., 2021b), and Mahmoud et al. combined frontal and lateral corneal images (Mahmoud and Mengash, 2021). Severity-classification tasks were predominantly multi-class. Classification tasks used stage 0–4 grading, six-category schemes including subclinical KC, and four-category schemes such as normal, mild, moderate and advanced. These targets differ in their reference criteria and case spectrum. Other tasks included continuous severity scores and exploratory severity characterization. Table 1 records the target and validation setting for each report.

**TABLE 1:** Severity assessment.

Study	Population and source	Target and model	Reported results
Muhsin et al. (2024)	644 eyes; Pentacam	Ramdom forest; Curvature and thinnest pachymetry; stages 0–4	Accuracy 98.6%
Gao et al. (2023)	488 eyes/281 patients; SCHWIND	Five maps; normal, mild, moderate, severe Lightweight keratoconus grading network	Weighted F1 0.895
Dhinakaran et al. (2023)	More than 2,000 original images; Pentacam	Maps; normal and 3 KC severity groups ResNet50 and EfficientNet	Accuracy 95.0%
Ambalgekar and Ashwini, (2023)	Dataset size insufficiently described	Topography; normal and three severity groups Inception V3	Accuracy 94.23%
Alyasseri et al. (2022)	Pentacam and CASIA datasets; units and reuse require caution	Clustering against normal and stages 1–4 Hybrid flower-pollination clustering	Accuracy 75.2%
Shashank et al. (2021)	2,071 augmented maps	Topography maps; normal and three severity groups Con-ker	Accuracy 94.6%
Malyugin et al. (2021)	852 eyes; pentacam	Seven parameters; normal, subclinical and stages 1–4 Discriminant models	Reported stage AUCs 0.95–1.00
Mahmoud and Mengash, (2021)	Confusion matrix datasets: 100 normal, 58 mild, 70 moderate, 40 severe	Corneal image reconstruction and classification CNN	Accuracy 97.8%,
Kamiya et al. (2021b)	349 eyes; TMS-4 placido topography	Absolute axial map; normal and stages 1–4 VGG-16	Grading accuracy 78.5%; stage 3 sensitivity 28.1%
Herber et al. (2021)	434 eyes/434 patients; corvis ST	40 biomechanical parameters; normal, mild, moderate, advanced Random forest	Grading accuracy 78%; linear discriminant 71%
Chen et al. (2021)	1,926 scans; pentacam; three centers	Four color-coded maps; five severity categories VGG16	External five-class AUC 0.859
Hallett et al. (2020)	237 samples; 29 pentacam parameters	Variational autoencoder + Gaussian mixture model; amsler–Krumeich stages 1–4	Clustering accuracy averaged 76.9% ± 3% over 20 repeats
Yousefi et al. (2018)	3,156 selected eyes; CASIA optical coherence tomography	Dimensionality reduction and clustering; four device-index-associated groups	Binary cluster sensitivity/specificity 97.7%/94.1%
Issarti et al. (2020)	812 subjects/eyes; pentacam HR	Neural-network continuous severity score	Suspect/normal sensitivity 85.2%, specificity 70.0%; 99.9% concerns binary detection
Bolarín et al. (2020)	169 eyes/169 people; a Sirius System^®^ tomographer	A multivariate logistic regression and an ordinal logistic regression model; healthy and RETICS I–V	Training accuracy 69.8% (95% CI 62.3%–76.6%)
Velázquez-Blázquez et al. (2020)	178 eyes / 178 subjects (74 healthy control, 104 KC) for observational comparative study; 41 eyes/ subjects for validation; Sirius tomography	Ordinal regression; healthy, RETICS I and II	Global accuracy 73% (95% CI 65%-79%)
Aatila et al. (2021)	3162 records from keratoconus dataset of Harvard Dataverse	Random forest with 10 selected features; four severity-related categories	Accuracy of 98% and 95% considering 2 and 4 keratoconus classes
Ghaderi et al. (2021)	450 eyes; pentacam; single clinic	Multilayer perceptron and neuro-fuzzy ensemble; normal and KC1–3	Four-class accuracy 98.2%
Muhsin et al. (2025b)	100 test samples; pentacam	Two-stage ensemble; stages 0–4	Validation accuracy 99.41%; unseen-test accuracy 99%
Tai et al. (2022)	31 eyes/31 KC patients; Corvis ST system	Clustering/decomposition; continuous severity referenced to TMS-4 indices	Symmetric-mode/normality correlation r = 0.92

AUC, area under the receiver operating characteristic curve; CDVA, corrected distance visual acuity; CI, confidence interval; CNN, convolutional neural network; KC, keratoconus; F1, harmonic mean of precision and recall; RETICS, thematic network for cooperative research in health grading system; VGG, Visual Geometry Group network; UK, United Kingdom; NZ, New Zealand; HR, high resolution.

Dataset size was reported in different units, including eyes, scans, augmented images, and records. For example, Chen et al. included 1,926 tomographic scans in a five-class Pentacam cohort (Chen et al., 2021), Kamiya et al. analyzed 349 eyes with TMS-4, Malyugin et al. evaluated 852 eyes with Pentacam (Malyugin et al., 2021), Herber et al. included 434 eyes with biomechanical measurements (Herber et al., 2021), Shashank et al. used 2,071 augmented topography images (Shashank et al., 2021), and Alyasseri et al. analyzed 5,430 Brazilian Pentacam records and 1,531 Japanese CASIA records (Alyasseri et al., 2022).

#### 3.1.2 Model application and feature strategies

Traditional ML approaches were primarily applied to structured tomographic and biomechanical parameters. Muhsin et al. compared random forests with logistic regression and support vector machines (Muhsin et al., 2024); while Malyugin et al. combined feature selection and dimensionality reduction with logistic regression and quadratic discriminant analysis (Malyugin et al., 2021); Herber et al. used random forests and linear discriminant analysis on biomechanical and pachymetric features (Herber et al., 2021); whereas Hallett et al. integrated unsupervised representation learning with a supervised multilayer perceptron classifier (Hallett et al., 2020). Alyasseri et al. further explored feature selection–assisted clustering using 50 parameters from Pentacam and CASIA devices (Alyasseri et al., 2022). Deep learning studies used direct analysis of topographic maps and corneal images. Gao et al. developed a ResNet18-based network with self-attention and multi-level feature fusion using five SCHWIND color-coded maps (Gao et al., 2023), Dhinakaran et al. compared ResNet50 and EfficientNet-B0 on color-coded corneal topography images (Dhinakaran et al., 2023). Ambalgekar et al. used an Inception V3 architecture on corneal topography maps, with MobileNet and CNNs reported as comparators (Ambalgekar and Ashwini, 2023), and both Chen et al. and Kamiya et al. used VGG16-based convolutional neural network (CNN) models, although with different map combinations and imaging platforms (Chen et al., 2021; Kamiya et al., 2021b). More specialized architectures were also developed to exploit richer spatial information: Mahmoud et al. combined frontal and lateral corneal images through 3D reconstruction (Mahmoud and Mengash, 2021), and Shashank et al. introduced a custom Con-Ker CNN integrating topography, pachymetry, and curvature maps (Shashank et al., 2021). These studies used both models based on handcrafted parameters and end-to-end image-based learning.

#### 3.1.3 Model performance and validation

Among traditional ML models, Muhsin et al. reported that random forests achieved very high five-class performance on three selected Pentacam parameters, with accuracy 0.9862, precision 0.9870, recall 0.9862, and F1-score 0.9866 (Muhsin et al., 2024). In the same dataset, logistic regression and support vector machines yielded lower performance, with logistic regression accuracy approximately 0.738 and support vector machine accuracy 0.800. Malyugin et al. reported an average area under the receiver operating characteristic curve (AUC) of 0.97 across normal, subclinical, and stages 1–4, with class-specific AUCs of 0.98 (normal), 0.95 (subclinical), 0.96–0.97 (stages 1–3), and 1.00 (stage 4) (Malyugin et al., 2021). Herber et al. obtained overall accuracies of 0.78 for random forests and 0.71 for linear discriminant analysis using Corvis-derived biomechanical and pachymetric parameters (Herber et al., 2021).

Deep learning–based tomographic or topographic models generally reported high discrimination. Gao et al. reported, on four-fold cross-validation, a weighted recall of 0.8955, weighted precision of 0.8998, weighted F1-score of 0.8950, and Cohen’s kappa of 0.9438 for their ResNet18-based attention/fusion network, compared with weighted recall 0.8709, precision 0.8783, F1-score 0.8605, and kappa 0.9271 for vanilla ResNet18 (Gao et al., 2023). Dhinakaran et al. reported an accuracy of 0.95 for ResNet50 and 0.95 for EfficientNet-B0 on Pentacam-based four-class grading (Dhinakaran et al., 2023). Ambalgekar et al. reported an overall accuracy of 0.9423 for Inception V3 on corneal topography maps, compared with 0.75 for MobileNet; other baseline CNNs (DenseNet121/169/201, Xception) were listed without explicit performance metrics (Ambalgekar and Ashwini, 2023). Shashank et al. reported an accuracy of 0.946 for their Con-Ker model, exceeding AlexNet (0.8478) and VGG19 (0.7705) (Shashank et al., 2021).

For color-map–based Pentacam CNNs, Chen et al. reported a five-class AUC of 0.859 for VGG16 using four color-coded maps in an external Iranian set of 532 scans (Chen et al., 2021). Kamiya et al. observed an overall accuracy of 78.5% and AUCs ranging from 0.888 to 0.997 across five grades; class-specific sensitivity, specificity, and accuracy varied, with high performance for grade 0 (sensitivity 0.988, specificity 0.944, accuracy 0.966) and lower sensitivity for grade 3 (0.281) despite preserved specificity (0.957) and accuracy (0.920) (Kamiya et al., 2021b). Mahmoud et al. reported an overall accuracy of 0.9780 for combined frontal and lateral images, but the displayed confusion-matrix diagonal gives 250/268 correct classifications (93.3%). This internal inconsistency limits interpretation of the reported accuracy (Mahmoud and Mengash, 2021).

Hallett et al. reported that an unsupervised clustering of 29 Pentacam parameters achieved a best-run accuracy of 82.4% and mean accuracy of 76.9% ± 3% over 20 runs for assigning eyes to Amsler–Krumeich stages 1–4, with stage-specific AUCs of 0.91, 0.87, 0.79, and 0.99, respectively (Hallett et al., 2020). Their supervised multilayer perceptron achieved a slightly lower overall accuracy of 73.9%, with AUCs of 0.92 (stage 1), 0.86 (stage 2), 0.83 (stage 3), and 0.91 (stage 4). Alyasseri et al., using flower-pollination-assisted K-means on 50 Pentacam/CASIA parameters, reported five-class accuracy 0.7520, precision 0.3501, recall 0.4770, F-score 0.4897, and purity 0.5364 (Alyasseri et al., 2022).

Issarti et al.’s Logik study compared its model with the Belin–Ambrósio enhanced ectasia display (BAD-D) and topographic keratoconus classification (TKC), and derived a continuous severity score, with an independent-center evaluation component (Issarti et al., 2020).

Validation settings varied across clustering and severity-scoring studies. Yousefi et al. interpreted unsupervised clusters against a device-derived ectasia index without a held-out grading test (Yousefi et al., 2018). Bolarín et al. reported 69.8% training accuracy for a morphogeometric classification using the grading system of the Thematic Network for Cooperative Research in Health (RETICS), whereas a related report of automatic scoring for early and mild KC evaluated a separate later same-center sample and obtained 71% accuracy with a wide 95% confidence interval of 55%–84% (Bolarín et al., 2020; Velázquez-Blázquez et al., 2020).

### 3.2 Keratoconus progression assessment and prediction

#### 3.2.1 Study characteristics and task definitions

Ten reports evaluated progression assessment or prediction (Table 2). Tomography-derived measurements were used as predictors or to generate progression labels in several studies (Jiménez-García et al., 2021; Kato et al., 2021; Shetty et al., 2021; Kundu et al., 2023). Shetty et al. used a dynamic labeling strategy based on Kmax change. The first visit served as the initial reference for each eye. Each subsequent visit was then labeled as “progression” or “no progression” according to whether Kmax had increased beyond a predefined threshold. Three thresholds were tested: 0.75 diopters (D), 1.0 D, and 1.25 D, corresponding to models A, B, and C. Once progression was detected, that visit became the new reference for evaluating later visits (Shetty et al., 2021). Kundu et al. prospectively collected clinical risk factors and built a random forest classifier on top of the progression labels generated by Shetty et al.‘s Pentacam-based model (Kundu et al., 2023). Kato et al. classified eyes by subsequent CXL receipt during follow-up ranging from 6 weeks to 8.6 years (Kato et al., 2021). This outcome reflects treatment policy as well as disease change and is not progression at a common fixed horizon. Jiménez-García et al. required at least three follow-up visits per patient and defined progression at the third visit as any of five tomographic indices (zonal maximum keratometry, anterior steep-meridian radius, anterior best-fit sphere, posterior mean radius, and the Logik score) changing beyond the 95% confidence interval from baseline in the direction consistent with worsening (Jiménez-García et al., 2021).

**TABLE 2:** Progression assessment and prognosis.

Study	Population and source	Target and model	Reported results
Future tomographic change — Jiménez-García et al. (2021)	629 eyes/421 patients after filtering; Pentacam	Time-delay network; five future tomographic indices; age plus prior measurements as inputs	Sensitivity 70.8%; specificity 80.6%
Future tomographic change — Hartmann et al. (2024)	570 eyes; patient count unclear; Pentacam; one center	MobileNet plus numerical variables; baseline prediction of ≥1 D Kmax increase; variable follow-up	Combined accuracy 83%, sensitivity 53%, specificity 95%; numerical-only accuracy 82%
Previously observed change — Shetty et al. (2021)	366 eyes/296 patients; 1,518 follow-up scans; Pentacam; one center	Random forest; Kmax changes ≥0.75, ≥1.00 or ≥1.25 D	AUCs 0.90, 0.91 and 0.93
Previously observed change — Kundu et al. (2023)	450 eyes/450 patients; prospective risk-factor collection	Clinical/ocular-surface factors; labels generated by tomographic classifier, based on ≥1 D Kmax change	Random forest; AUC 0.812
Previously observed change — Kamiya et al. (2021a)	218 eyes/218 patients; CASIA AS-OCT; two hospitals	Xception plus age; classification of progression documented during the preceding 12 months	Age-adjusted accuracy 84.9%; sensitivity 95.5%; specificity 58.1%
Early detection — Reddy et al. (2022)	Development: 385 eyes/351 patients; evaluation: 1,331 eyes/828 patients; Oculyzer	Paired-map convolutional networks; serial change before ≥1 D increase in flat or steep keratometry	Progression signal detected a mean 11.1 months earlier
Longitudinal phenotyping — Cao et al. (2023)	1,017 KC eyes and 128 controls; Pentacam	Hierarchical clustering of 6-month tomographic changes; fast, slow and minimal-change phenotypes	Three clusters; 39 curvature variables distinguished groups; similar clusters at 6–18 months
Longitudinal stability — Han et al. (2023)	163 eyes/127 patients; Pentacam; mean follow-up 55.5 months	Posterior-elevation stability; logistic regression and support vector machine using three tomographic variables	AUC: logistic regression 0.780; support vector machine 0.659
Treatment-policy endpoint — Kato et al. (2021)	274 eyes/158 patients; Pentacam	DL model; actual CXL receipt over 6 weeks–8.6 years	Fivefold AUC 0.814; sensitivity 77.8%; specificity 69.6%
Treatment-policy endpoint — Cohen-Tayar et al. (2025)	1,218 tests (677 eyes/481 patients); serial subset 819 scans of 276 eyes; Pentacam; one center	Boosted decision trees; subsequent CXL referral; single versus serial examination inputs	AUC: 0.59 single, 0.75 serial; serial precision 0.32, recall 0.67

DL, deep learning; AS-OCT, anterior-segment optical coherence tomography; AUC, area under the receiver operating characteristic curve; CXL, corneal cross-linking; D, diopters; KC, keratoconus; Kmax, maximum keratometry.

As for sample size, Kundu et al. analyzed 450 eyes (322 progressors, 128 non-progressors) (Kundu et al., 2023). Shetty et al. included 366 eyes (296 patients) and 1,884 Pentacam scans, with the number of progression samples varying by model (224 for model A, 161 for model B, and 125 for model C). Kato et al. included 274 eyes (158 patients), of which 90 were classified as CXL+ and 184 as CXL− (Kato et al., 2021). Jiménez-García et al. drew on a larger source population, but filtering produced 629 eyes from 421 patients for the reported sequence construction (Jiménez-García et al., 2021). Kamiya et al. studied 218 eyes from 218 patients at two hospitals using AS-OCT maps and progression documented during the preceding 12 months (Kamiya et al., 2021a).

#### 3.2.2 Model application and feature strategies

Models used tomographic maps, structured tomographic measurements, or clinical and systemic predictors. Hartmann et al. compared combined image and numerical inputs with numerical-only and image-only models (Hartmann et al., 2024). Shetty et al. used tomographic curvature, pachymetric, irregularity and optical-aberration features in a random forest classifier under three Kmax-threshold scenarios (Shetty et al., 2021). Kundu et al. used ocular-surface risk factors (allergy history, eye rubbing, and daily computer use time) and systemic markers such as immunoglobulin E and vitamins D and B12, as predictors for a random forest classifier (Kundu et al., 2023). Kato et al. trained a VGG16 CNN on three Pentacam color-coded maps (axial curvature, pachymetry, and a combined map), supplemented by age as an additional predictor, to classify eyes as CXL+ or CXL− (Kato et al., 2021). Jiménez-García et al. used age and five tomographic indices in a time delay neural network, capturing temporal information from multiple visits (Jiménez-García et al., 2021). The time-delay study split visit triplets for development and testing; independence at the eye or patient level was not established.

#### 3.2.3 Model performance and validation

The performance and validation results are interpreted separately for four endpoint groups: future tomographic change, previously observed or algorithm-defined change, early detection or longitudinal phenotyping, and subsequent CXL referral or receipt.

For future tomographic change, Jiménez-García et al.’s time-delay neural network used prior visits and reported sensitivity of 70.8%, specificity of 80.6%, positive predictive value of 71.4% and negative predictive value of 80.2% at the third visit (Jiménez-García et al., 2021). Hartmann et al. reported accuracy of 0.83 for combined inputs versus 0.82 for numerical variables alone and 0.77 for images alone (Hartmann et al., 2024).

For previously observed or algorithm-defined change, Shetty et al. reported AUCs of 0.90, 0.91 and 0.93 for Kmax thresholds of 0.75, 1.00 and 1.25 D, respectively; corresponding sensitivities were 85%, 86% and 89%, specificities 82%, 82% and 81%, accuracies 83%, 88% and 91%, and F1-scores 82%, 88% and 90% (Shetty et al., 2021). Kundu et al. reported an AUC of 0.812 (95% confidence interval 0.711–0.897) against algorithm-derived labels (Kundu et al., 2023). Kamiya et al. reported age-adjusted accuracy of 0.849 for progression documented during the preceding 12 months (Kamiya et al., 2021a).

For early detection and longitudinal phenotyping, Reddy et al. detected a progression signal a mean of 11.1 months before a conventional 1 D change in flat keratometry (K1) or steep keratometry (K2) in a cohort composed of eventual progressors (Reddy et al., 2022). Cao et al. clustered 6-month tomographic changes into fast, slow and minimal-change phenotypes, which describes longitudinal trajectories (Cao et al., 2023). Han et al. modeled posterior-elevation stability over variable follow-up (Han et al., 2023).

For treatment-policy endpoints, Kato et al. predicted subsequent CXL receipt over 6 weeks to 8.6 years (AUC 0.814, sensitivity 77.8%, specificity 69.6%) (Kato et al., 2021), and Cohen-Tayar et al. predicted subsequent CXL referral, with AUC increasing from 0.59 for a single examination to 0.75 for serial examinations (Cohen-Tayar et al., 2025).

### 3.3 Keratoconus surgical outcome prediction

#### 3.3.1 Study characteristics and task definitions

Eleven reports evaluated ML models for predicting outcomes after KC treatment (Table 3). These include corneal cross-linking, intracorneal ring segments (ICRS), and keratoplasty. Across the 11 reports, targets included postoperative visual and refractive outcomes, corneal thickness, progression after CXL, composite graft outcomes, graft rejection, and postoperative corneal maps. Follow-up horizons varied by procedure and endpoint (Table 3).

**TABLE 3:** Prediction of outcomes after KC treatment.

Study	Population and source	Target and model	Reported results
CXL — continuous visual/keratometric outcome — Liu et al. (2023)	Development: 277 eyes/195 patients; later validation: 43 eyes/35 patients; Pentacam	XGBoost; changes in corrected distance visual acuity and Kmax 2 years after CXL	Temporal-validation R^2^: acuity 0.8956; Kmax 0.8382
CXL — thickness outcome — Statsenko et al. (2025)	131 eyes/107 patients; 796 observations; Oculyzer II; one center	LightGBM; minimum/central corneal thickness after CXL; mean follow-up 14 months	Test root mean squared error: minimum thickness 19.22 µm; central thickness 19.68 µm
CXL — progression event — Wan et al. (2025)	95 eyes/69 patients; Pentacam and Corvis ST; one center	Kmax and surface-variance nomogram; progression after accelerated CXL using the study’s E-stage criterion; 3–22 months	Progression: 51.4% high-risk versus 16% low-risk
ICRS — optical/visual regression — Mehdizadeh Dastjerdi et al. (2023)	Eight task datasets, each ≥40 input–target pairs; ring-treated KC	Neural networks; topographic and visual parameters at 6 and 12 months	leave-one-out cross-validation errors: 6.82% (topographic indices) and 7.65% (visual characteristics).
ICRS — optical/visual regression — Perez et al. (2026)	120 eyes/102 patients; MS-39 maps and clinical data; one center	CatBoost/XGBoost; visual acuity, keratometry and asphericity after ring implantation	CatBoost acuity R^2^ 0.59, MAE 0.07 logMAR; mean keratometry MAE 1.08 D
ICRS — nomogram comparison — Lyra et al. (2018)	209 eyes/160 patients; Pentacam	Linear regression; post-ICRS Q and mean K; median 28 months (5–72)	MAE Q 0.19 vs. nomogram 0.30; mean K 1.18 D vs. 1.27 D
ICRS — treatment-planning comparison — Fariselli et al. (2020)	75 successful cases for training; pilot 20 vs. 20 eyes	Neural-network ring planning; 3-month pilot outcomes	Final decimal CDVA 0.73 vs. 0.62 (nomogram)
ICRS — exploratory postoperative outcome — Hassan Musa et al. (2024)	Postoperative cohort size unclear; separate diagnostic dataset of 900 images (450 KC/450 normal)	RNN/LSTM; five visual parameters as inputs; visual outcomes at 6 and 12 months after KeraRing or MyoRing implantation	Test R at 6/12 months: KeraRing 0.949/0.923; MyoRing 0.920/0.909. Average prediction errors: 9.82%/9.29% and 11.46%/7.47%, respectively
Keratoplasty — composite outcome — Kundu et al. (2025)	250 eyes/250 patients; stage 4 KC; Pentacam and clinical variables	Random forest; favorable DALK composite incorporating graft clarity, sutures, vascularization and visual acuity; ≥5 years	AUC 0.957; classification accuracy 91.4%. Highest-ranked predictors: systemic allergy, serum IgE, eye rubbing, vitamin D
Keratoplasty — rejection association — Feizi et al. (2024)	1,214 eyes/985 KC patients; PK and DALK	ANN, SVM, gradient boosting, extra trees and random survival forests; recipient, donor, operative and postoperative variables	C-statistics 0.664–0.675; test accuracies 74.2%–76.3% across five models
Map generation — image similarity — Fırat et al. (2022)	125 eyes/96 patients; Pentacam	U-Net-based 2D regression; preoperative images plus pre/postoperative interpolated images; 6-month post-CXL maps	Mean SSIM 0.8266; pixel-level RMSE 0.134; PSNR 65.850

AUC, area under the receiver operating characteristic curve; logMAR, logarithm of the minimum angle of resolution; CDVA, corrected distance visual acuity; CXL, corneal cross-linking; D, diopters; DALK, deep anterior lamellar keratoplasty; PK, penetrating keratoplasty; ICRS, intracorneal ring segments; IgE, immunoglobulin E; KC, keratoconus; Kmax, maximum keratometry; MAE, mean absolute error; Q, corneal asphericity; R^2^, coefficient of determination; SSIM, structural similarity index. ANN, artificial neural network; SVM, support vector machine; RMSE, root mean square error; PSNR, peak signal-to-noise ratio.

#### 3.3.2 Model application and feature strategies

For CXL outcomes, Liu et al. used XGBoost to predict changes in visual acuity and Kmax (Liu et al., 2023), Statsenko et al. used LightGBM to estimate corneal thickness (Statsenko et al., 2025), and Wan et al. evaluated a nomogram incorporating Kmax and surface variance for post-CXL progression (Wan et al., 2025). ICRS studies used several input and modeling strategies. Mehdizadeh Dastjerdi et al. developed separate feed-forward artificial neural networks (ANNs) for 6- and 12-month outcomes after KeraRing or MyoRing implantation: topographic models used astigmatism, K-reading, and pachymetry together with ring diameter and thickness, while visual models used five preoperative visual parameters together with the same ring characteristics (Mehdizadeh Dastjerdi et al., 2023). Hassan Musa et al. described a recurrent neural network with long short-term memory (RNN/LSTM), using uncorrected visual acuity, sphere, astigmatism, axis, and best-corrected visual acuity as inputs for postoperative visual prediction (Hassan Musa et al., 2024). Perez et al. evaluated CatBoost and XGBoost using MS-39 maps and clinical data (Perez et al., 2026), Lyra et al. used linear regression for ring selection (Lyra et al., 2018), and Fariselli et al. used an ANN to guide ICRS planning (Fariselli et al., 2020). For keratoplasty, Kundu et al. combined preoperative clinical factors and Pentacam parameters in a random forest classifier (Kundu et al., 2025), while Feizi et al. compared five models using recipient, donor, operative, and postoperative variables (Feizi et al., 2024). For postoperative map generation, Fırat et al. analyzed paired images from 125 eyes of 96 patients, replaced the U-Net classification output with a regression layer and used preoperative images together with images interpolated between preoperative and postoperative maps (Fırat et al., 2022).

#### 3.3.3 Model performance and validation

For CXL-related outcomes, Liu et al. predicted changes in corrected distance visual acuity (CDVA) and Kmax 2 years after CXL, with temporal-validation coefficients of determination (R^2^) of 0.8956 and 0.8382 in a later same-center cohort (Liu et al., 2023). Statsenko et al. reported test root mean square errors (RMSEs) of 19.22 µm for minimum corneal thickness and 19.68 µm for central corneal thickness after CXL (Statsenko et al., 2025), while Wan et al. reported post-CXL progression in 51.4% of the high-risk group versus 16.0% of the low-risk group, defined using the study’s E-stage criterion over 3–22 months (Wan et al., 2025).

For ICRS planning and postoperative optical or visual outcomes, Mehdizadeh Dastjerdi et al. reported mean absolute percentage errors of 7.29% for topographic indices and 8.60% for visual parameters across the full datasets; the corresponding mean errors in leave-one-out cross-validation were 6.82% and 7.65% (Mehdizadeh Dastjerdi et al., 2023). Perez et al. predicted visual acuity, keratometry and asphericity after ring implantation, with an acuity R^2^ of 0.59 and mean-keratometry mean absolute error (MAE) of 1.08 D (Perez et al., 2026). Lyra et al. compared a regression model with the manufacturer’s nomogram: asphericity MAE was 0.19 versus 0.30 and mean-keratometry MAE 1.18 versus 1.27 D (Lyra et al., 2018). A similar comparative approach was taken by Fariselli et al., who compared ANN-guided and manufacturer-nomogram-guided ICRS implantation in two groups of 20 eyes. At 3 months, mean decimal CDVA improved from 0.60 to 0.73 in the ANN group and from 0.54 to 0.62 in the nomogram group, with significantly better postoperative CDVA in the ANN group (P < 0.05) (Fariselli et al., 2020). Hassan Musa et al. reported test-set correlations of R = 0.949 and 0.923 for predictions of visual outcomes at 6 and 12 months after KeraRing implantation, respectively, and R = 0.920 and 0.909 after MyoRing implantation. The reported average prediction errors were 9.82% and 9.29% for KeraRing and 11.46% and 7.47% for MyoRing at the corresponding time points (Hassan Musa et al., 2024).

For keratoplasty outcomes, Kundu et al. used a random forest model to classify favorable versus unfavorable outcomes after deep anterior lamellar keratoplasty (DALK) in 250 eyes with at least 5 years of follow-up, based on graft status and visual acuity changes. Combining preoperative clinical factors and Pentacam parameters, the model achieved an AUC of 0.957 and a reported classification accuracy of 91.4% using leave-one-out cross-validation. Systemic allergy, serum IgE, eye rubbing, and vitamin D were the highest-ranked predictors (Kundu et al., 2025). Whereas this study focused on the long-term observation of visual acuity and graft status, Feizi et al. examined graft rejection. They evaluated five ML models for graft rejection in 1,214 eyes undergoing penetrating keratoplasty (PK) or DALK. With an 80:20 training–test split, the models yielded C-statistics of 0.664–0.675 and test accuracies of 74.2%–76.3%. Keratoplasty technique was identified as a predictor across all five models, while corticosteroid treatment duration and suture-related factors were identified by several models (Feizi et al., 2024).

For postoperative map generation, Fırat et al. reported a mean structural similarity index (SSIM) of 0.8266, a pixel-level RMSE of 0.134, and a peak signal-to-noise ratio (PSNR) of 65.850 when comparing generated maps with observed 6-month post-CXL maps (Fırat et al., 2022).

## 4 Discussion

This review identifies different levels of development across ML applications for keratoconus severity classification, progression prediction, and postoperative outcome prediction. Severity classification has the broadest evidence base, with many models reporting high internal discrimination from corneal imaging. Progression and postoperative studies address more directly actionable questions, but their targets range from observed morphological change to treatment receipt, visual outcomes, and graft complications. These differences are central to interpreting performance: a model that reproduces an existing stage, detects change, or estimates a future outcome supports a different clinical decision. Across the three domains, the main unresolved question is how much useful information models add to conventional assessment when applied to new patients and clinical settings.

For severity classification, the consistent performance of models using tomographic indices, biomechanical measurements, and color-coded maps indicates that these inputs capture information relevant to established disease stages. However, the choice of reference classification strongly shapes what a model learns. When labels are assigned using Amsler–Krumeich or ABCD criteria (Krumeich et al., 1998; Belin and Duncan, 2016), agreement primarily demonstrates reproduction of that framework; it does not establish improved assessment of visual function or subsequent deterioration. High overall accuracy can also obscure difficulty distinguishing adjacent stages or underrepresented categories. Comparisons are therefore most informative when models use the same patients and reference labels, report errors by stage, and include conventional indices or a simpler statistical model as comparators. Such comparisons would clarify whether image-based learning adds useful information beyond measurements already available to clinicians.

For progression, the distinction between detecting change and forecasting it is particularly important. Studies using serial examinations can characterize an evolving cornea, whereas baseline prediction must estimate an outcome that has not yet occurred. CXL referral or receipt introduces another target, reflecting clinical practice and treatment availability as well as disease status. Variation in Kmax thresholds, composite definitions, and follow-up intervals further limits comparison across cohorts (Gil et al., 2025; Henriquez et al., 2025). The most useful future evaluations would define the prediction time and horizon, distinguish untreated from post-treatment trajectories, and establish that observed change exceeds measurement variability (Zheng et al., 2017). For monitoring decisions, the practical value of a forecast depends on whether it improves identification of eyes needing closer review or intervention beyond routine longitudinal assessment.

Postoperative prediction is similarly procedure- and outcome-specific (Yüksel et al., 2024; Omar and Elhiny, 2025). ICRS studies estimate optical or visual changes, while model-guided planning also concerns the choice of implant. Fariselli et al.'s comparison with a manufacturer nomogram provides preliminary evidence linking ANN-guided planning to postoperative CDVA, although the small, nonrandomized groups and differing use of concomitant CXL limit attribution of the difference to the model (Fariselli et al., 2020). For keratoplasty, Kundu et al.'s high AUC concerns a composite favorable versus unfavorable DALK outcome (Kundu et al., 2025), whereas Feizi et al. examined graft rejection using a broader set of perioperative variables (Feizi et al., 2024). Continuous outcome models also require absolute prediction errors in clinically interpretable units.

Postoperative map generation provides a complementary way to represent corneal change, but its evaluation must reflect how a model would be used. Fırat et al. reported similarity between generated and observed 6-month post-CXL maps; their augmented inputs were interpolated from both preoperative and postoperative images (Fırat et al., 2022). This design demonstrates map-generation performance under the reported experimental conditions, while prediction using only information available before treatment remains to be established. The same timing principle applies to clinical predictors: postoperative events may inform follow-up risk assessment, but they cannot support a decision made before those events occur. Defining the intended decision point is therefore essential to evaluating postoperative models.

Several shared design issues constrain generalizability. Much of the evidence comes from retrospective cohorts with limited center and device diversity, so performance may depend on referral patterns, scan quality, acquisition settings, and treatment practices. Patient-level separation is especially important when datasets contain fellow eyes, repeated visits, or augmented images; these observations are not independent substitutes for additional patients. Preprocessing and model selection must also remain within the development data. A later cohort from the same center provides useful temporal validation, but independent centers and devices test different aspects of transportability. These concerns favor evaluation of a fixed model in clearly defined external cohorts, with errors and failures reported across relevant disease stages and patient groups, rather than reliance on internal discrimination alone.

Clinical evaluation should connect predictive performance to the decision the model is intended to support. Calibration and uncertainty matter when estimated risks guide monitoring or intervention, while absolute errors are needed for continuous postoperative outcomes (Van Calster et al., 2019). Prospective workflow studies can then determine whether predictions improve decisions, including missed progression and unnecessary intervention, compared with usual assessment (Vasey et al., 2022). A practical research priority is to establish comparable clinical endpoints and evaluation datasets before expanding model complexity. Multimodal inputs may add complementary information, but their incremental value should be tested against tomography-based and simpler models in the same patients (Cao et al., 2025). Clear reporting of cohort selection, data partitions, outcome definitions, and model evaluation would make these comparisons more informative and support reproducibility (Collins et al., 2024; Tejani et al., 2024; Shao et al., 2025; Yang et al., 2025; Yuan et al., 2025).

This review has three main limitations. First, substantial heterogeneity in study populations, imaging inputs, outcome definitions, follow-up periods, and validation strategies precluded a meaningful pooled estimate of performance or a reliable ranking of algorithms. Second, incomplete reporting of patient counts, data partitions, and predictor timing in some studies limited our ability to assess the independence of evaluation data and the applicability of reported results (Moons et al., 2025). Third, the limited availability of external validation and prospective clinical evaluation constrained conclusions about generalizability and clinical benefit.

## 5 Conclusion

In summary, ML and DL show clear potential for keratoconus severity classification, progression assessment and prediction, and postoperative outcome forecasting. However, current evidence is limited by single-center designs, scarce external validation, and limited assessment of clinical utility. Future work should prioritize standardized definitions, multicenter validation, multimodal integration, and real-world evaluation to translate technical performance into clinically useful decision support.
